# Supplementary material for: Identification of Novel Circular RNAs of the Human Protein Arginine Methyltransferase 1 (PRMT1) Gene, Expressed in Breast Cancer Cells
Source: Genes (Basel). 2022 Jun 24;13(7):1133. doi: 10.3390/genes13071133 (PMC9316507; doi:10.3390/genes13071133)
Supplement: Supplementary file 1 [file genes-13-01133-s001.zip › Supplementary Tables/Table S1.pdf]

Table S1. Characteristics of the four breast cancer cell lines that were used in the study.

Cell line	Disease histology	Morphology	Molecular subtype	Tumor source	ER status [1]	PR status [2]	HER2 + status[3]	Some Mutated Genes
BT-20	Carcinoma	Epithelial	Triple-negative, Luminal Androgen Receptor (LAR)	Primary	-	-	-	*CDKN2A*, *PIK3CA*, *TP53*
MCF-7	Adenocarcinoma	Epithelial	Luminal A	Metastasis, pleural effusion	+	+	-	*CDKN2A*, *GATA3*, *PIK3CA*, *TP53*
MDA-MB-231	Adenocarcinoma	Epithelial	Triple-negative, Mesenchymal Stem-Like (MSL)	Metastasis, pleural effusion	-	-	-	*BRAF*, *CDKN2A*, *KRAS*, *NF2*, *TP53*
MDA-MB-468	Adenocarcinoma	Epithelial	Triple-negative, Basal-Like 1 (BL1)	Metastasis, pleural effusion	-	-	-	*PTEN*, *RB1*, *SMAD4*, *TP53*

[1] estrogen receptor, [2] progesterone receptor, [3] human epidermal growth factor receptor 2
