# Supplementary material for: Identification of Novel Circular RNAs of the Human Protein Arginine Methyltransferase 1 (PRMT1) Gene, Expressed in Breast Cancer Cells
Source: Genes (Basel). 2022 Jun 24;13(7):1133. doi: 10.3390/genes13071133 (PMC9316507; doi:10.3390/genes13071133)
Supplement: Supplementary file 1 [file genes-13-01133-s001.zip › Supplementary Tables/Table S2.pdf]

Table S2. Primers used in first- and second-round PCR assays for the selective amplification of *PRMT1* circular variants and the main variant of *GAPDH*.

Direction	Name	Sequence (5'→3')	Length (nt[1])	T_m (°C)	T_a (°C)
F	PRMT1_5F	CGGCACCTCTTCAAGGACAA	20	61	59
R	PRMT1_5R	ATGAAACATGGAGTTGCGGT	20	58	
F	PRMT1_5Fin	GGCATCCTCTGCATGTTTGC	20	61	60
R	PRMT1_5Rin	CCTCGTCCTTCAGCATCTC	19	59	
F	PRMT1_7F	GAGTCCATGCTCAACACCGT	20	61	61
R	PRMT1_7R	GAAGAGGCAGTAGCCCATCC	20	61	
F	PRMT1_7Fin	CTATGCCCGGGACAAGTGG	19	62	61
R	PRMT1_7Rin	TCGCTGATGATGATGTCCACC	21	60	
F	GAPDH Ex2 F	CCACATCGCTCAGACACCAT	20	60	60
R	GAPDH Ex4 R	TGACAAGCTTCCCGTTCTCA	20	58	

[1] nucleotides
