# Supplementary material for: Identification of Novel Circular RNAs of the Human Protein Arginine Methyltransferase 1 (PRMT1) Gene, Expressed in Breast Cancer Cells
Source: Genes (Basel). 2022 Jun 24;13(7):1133. doi: 10.3390/genes13071133 (PMC9316507; doi:10.3390/genes13071133)
Supplement: Supplementary file 1 [file genes-13-01133-s001.zip › Supplementary Tables/Table S3.pdf]

Table S3. The full list of miRNAs that are predicted to bind to the novel *PRMT1* circRNAs, according to the miRDB database.

PRMT1 circRNA	Target miRNA	Prediction score[1]
circ-PRMT1-1	hsa-miR-6754-3p	80
	hsa-miR-494-3p	76
	hsa-miR-1306-5p	71
	hsa-miR-412-3p	65
	hsa-miR-4433b-3p	64
	hsa-miR-4756-3p	60
	hsa-miR-3973-3p	52
	hsa-miR-6852-3p	51
	hsa-miR-648-5p	50
circ-PRMT1-2	hsa-miR-6754-3p	77
	hsa-miR-494-3p	76
	hsa-miR-1306-5p	71
	hsa-miR-412-3p	67
	hsa-miR-4433b-3p	63
	hsa-miR-4756-3p	60
	hsa-miR-3973-3p	52
	hsa-miR-3682-3p	51
	hsa-miR-6852-3p	51
circ-PRMT1-3	hsa-miR-494-3p	76
	hsa-miR-6754-3p	68
	hsa-miR-4433b-3p	63
	hsa-miR-6883-5p	60
	hsa-miR-6785-5p	60
	hsa-miR-4728-5p	60
	hsa-miR-149-3p	60
	hsa-miR-4756-3p	59
	hsa-miR-412-3p	57
	hsa-miR-3973-3p	52
	hsa-miR-6852-3p	51
circ-PRMT1-4	hsa-miR-494-3p	76
	hsa-miR-6754-3p	68
	hsa-miR-4433b-3p	63
	hsa-miR-4756-3p	60
	hsa-miR-412-3p	57
	hsa-miR-3973-3p	52
	hsa-miR-6852-3p	51
circ-PRMT1-5	hsa-miR-494-3p	75
	hsa-miR-6754-3p	67
	hsa-miR-4433b-3p	62
	hsa-miR-4745-3p	61
	hsa-miR-4756-3p	59
	hsa-miR-1538-3p	57
	hsa-miR-412-3p	56
	hsa-miR-3973-3p	51
	hsa-miR-6852-3p	50
circ-PRMT1-6	hsa-miR-494-3p	78

	hsa-miR-6754-3p	73
	hsa-miR-4756-3p	65
	hsa-miR-412-3p	62
	hsa-miR-2467-3p	50
circ-PRMT1-7	hsa-miR-494-3p	76
	hsa-miR-1306-5p	66
	hsa-miR-4756-3p	64
	hsa-miR-4433b-3p	63
	hsa-miR-3973-3p	52
	hsa-miR-6852-3p	51
circ-PRMT1-8	hsa-miR-4745-3p	65
	hsa-miR-1538-3p	62
	hsa-miR-3682-3p	57
	hsa-miR-4646-5p	53
	hsa-miR-204-3p	53
	hsa-miR-4314-5p	52
circ-PRMT1-9	hsa-miR-4696-5p	86
	hsa-miR-588-5p	71
	hsa-miR-4701-5p	71
	hsa-miR-1827-3p	65
	hsa-miR-4745-3p	63
	hsa-miR-4713-5p	62
	hsa-miR-1538-3p	59
	hsa-miR-4283-5p	51
	hsa-miR-6813-5p	50
	hsa-miR-6085-3p	50
	hsa-miR-4646-5p	50
	hsa-miR-204-3p	50

[1]The prediction score is on a scale up to 100.
