# Supplementary material for: Identification of Novel Circular RNAs of the Human Protein Arginine Methyltransferase 1 (PRMT1) Gene, Expressed in Breast Cancer Cells
Source: Genes (Basel). 2022 Jun 24;13(7):1133. doi: 10.3390/genes13071133 (PMC9316507; doi:10.3390/genes13071133)
Supplement: Supplementary file 1 [file genes-13-01133-s001.zip › Supplementary Tables/Table S5.pdf]

Table S5. The results from the Kyoto Encyclopedia of Genes and Genomes (KEGG) pathway analysis regarding the miRNA-targeted mRNAs, whose expression is predicted to be affected by the 9 *PRMT1* circRNAs.

***PRMT1* circRNA**	**Target miRNA**	**miRNA-targeted mRNA**	**KEGG pathway**
circ-PRMT1-1	miR-494-3p	*STT3B*	Metabolic pathways
	miR-6754-3p	*ATP5F1E*	
circ-PRMT1-2	miR-494-3p	*STT3B*	
	miR-6754-3p	*ATP5F1E*	
circ-PRMT1-3	miR-494-3p	*STT3B*	
	miR-6754-3p	*ATP5F1E*	
circ-PRMT1-4	miR-494-3p	*STT3B*	
	miR-6754-3p	*ATP5F1E*	
circ-PRMT1-5	miR-494-3p	*STT3B*	
	miR-6754-3p	*ATP5F1E*	
circ-PRMT1-6	miR-494-3p	*STT3B*	
	miR-6754-3p	*ATP5F1E*	
circ-PRMT1-7	miR-494-3p	*STT3B*	Metabolic pathways
	miR-1306-3p	*PDHA1*	
			Glucagon signaling pathway
		PPP3CC	
			Calcium signaling pathway
circ-PRMT1-8	miR-1538-3p	*CERS1*	Metabolic pathways
		GNAS	Calcium signaling pathway; cAMP signaling pathway; glucagon signaling pathway; pathways in cancer
		RAI1	Calcium signaling pathway; cAMP signaling pathway
	miR-4745-3p	*GNAS*	Calcium signaling pathway; cAMP signaling pathway; glucagon signaling pathway; pathways in cancer
circ-PRMT1-9	miR-588-5p	*COL4A4*	Pathways in cancer
		MGAT1	Metabolic pathways
		SPTLC2	
	miR-4696-5p	*MAX*	Pathways in cancer
		RARB	
		VAV3	cAMP signaling pathway
